# Herpes zoster stromal disciform keratitis associated with endotheliitis in a 35-week pregnant woman

**DOI:** 10.1093/jscr/rjaf219

**Published:** 2025-04-12

**Authors:** Jonas Akambase, Cadric Gunaratnam, Brian Todd, Stephen O'Hagan

**Affiliations:** Ophthalmology Department, Cairns Hospital, 165 the Esplanade, Cairns, QLD 4878, Australia; Ophthalmology Department, Cairns Hospital, 165 the Esplanade, Cairns, QLD 4878, Australia; Ophthalmology Department, Cairns Hospital, 165 the Esplanade, Cairns, QLD 4878, Australia; Ophthalmology Department, Cairns Hospital, 165 the Esplanade, Cairns, QLD 4878, Australia

**Keywords:** varicella zoster virus, endotheliitis, keratitis, Wessley ring, pregnancy

## Abstract

Herpes zoster keratitis is a common manifestation of the varicella zoster virus. The active virus can cause epithelial keratitis early in the disease course, meanwhile later in the disease course, an inflammatory response can cause stromal or endothelial keratitis. A middle-aged pregnant woman exhibited classic ocular signs associated with Herpes’s stromal disciform keratitis of the left eye, including focal disk-shaped edema, descemet’s folds, keratic precipitates, diminished corneal sensations, and Wessley’s immune ring. The patient was symptomatic with severe left eye pain, lacrimation, photophobia, and hand-motion visual acuity. To the best of our knowledge, this case represents the first report and an interesting favorable outcome of herpes zoster stromal disciform keratitis management in an Indigenous Australian.

## Introduction

Varicella-zoster virus (VZV) is a DNA virus in the herpesvirus family [1]. It is a common vector for illness such as chicken pox most commonly occurs in childhood and is spread by airborne, droplet, and contact transmission. Herpes zoster results from reactivation of the latent VZV within a sensory nerve ganglion, often presenting decades after the initial infection as shingles with advancing age and in patients with immunosuppression owing to other diseases or factors [2]. The reactivation of the latent VZV could result variation of herpes zoster that can cause a variety of ocular complications and requires urgent treatment [2].

Primary VZV infection during pregnancy has been associated with severe maternal illness and abnormalities among infants whose mothers had varicella during the first trimester. There were no abnormalities among infants whose mothers had second or third trimester varicella or among infants whose mothers had zoster [3]. Determining a woman’s serostatus prior to pregnancy is advised, as effective vaccines are now available and should be administered to non-pregnant seronegative women of child-bearing age [4].

Corneal involvement of herpes zoster infection includes both epithelial and stromal keratitis and endotheliitis [5]. To the best of our knowledge, stromal disciform keratitis associated with endotheliitis, caused by herpes zoster is very rare and has only been reported among cytomegalovirus (CMV) and herpes simplex virus (HSV) patients [5]. In this report, we describe a case of stromal disciform keratitis associated with endotheliitis caused by herpes zoster in a pregnant woman. In this paper, the word disciform is used interchangeably with disk-shaped or coin-shaped.

## Case report

A middle-aged Australian indigenous pregnant woman presented to the eye clinic with painful left red eye. The patient was 35-week pregnant and has a background of gestational diabetes.

**Figure 1:**
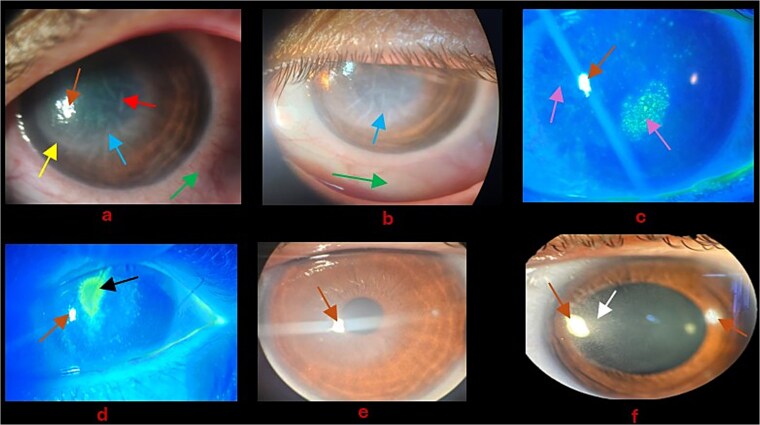
(a) Day 1: Blue arrow—Disk-shaped edema with endotheliitis; green arrow—Ciliary conjunctival injection; yellow arrow—Wessley immune ring; red arrow—DMF; Brown arrow—Corneal reflection. (b) Day 2 post admission: Blue arrow—Disk-shaped edema with endotheliitis remained unchanged; green arrow—Ciliary conjunctival injection improved. (c) Purple arrow—Punctate epithelial erosions; Brown arrow—Corneal reflection. (d) Black arrow—Corneal epithelial defect; Brown arrow—Corneal reflection. (e) Significant improvement of corneal edema and endotheliitis; Brown arrow—Corneal reflection. (f) Dilated pupils for fundus exam; white arrow—Stromal scar; Brown arrow—Corneal reflection.

Prior to presenting to the eye clinic, the patient saw a local GP and her eye condition was treated with Chloramphenicol eye drops four times daily and eye lubricants for 2 weeks with no improvement but worsening of the eye condition. Thus, the patient was referred to the outpatient ophthalmology clinic. She complained of left eye pain, loss of vision, lacrimation and light sensitivity. The best corrected visual acuity (BCVA) for the left eye was hand motion (HM) and 6/6 for the right eye. The intraocular pressures (IOP) were 11 mmHg and 6 mmHg for the right and left eye respectively. For the affected eye (left eye), marked ciliary conjunctival injection, and focal disk-shaped edema, descemet’s folds (DMF), keratic precipitates (KPs), diminished corneal sensations and Wessley’s immune ring (Shown in Fig. 1a). Fluorescein stain showed punctate epithelial erosions as indicated in Fig. 1c.

At the time of assessment, it was challenging to differentiate the clinical presentation from acanthamoeba infection. Cornea scraping, swaps for microscopy, culture and sensitivity (MCS), and polymerase chain reaction (PCR) for HSV, Varicella zoster virus (VZV), CMV, and adenovirus were performed. Herpes or CMV keratitis was the working diagnosis. However, acanthamoeba was also in the differential diagnosis. Distinguishing herpes and CMV keratitis from acanthamoeba is important due to the difference in prognosis and treatment.

The patient was admitted and started on acyclovir ointment 3% five times daily, Prednefrin forte eye drops every two hours [Prednisolone acetate 10 mg/mL (1%) and phenylephrine hydrochloride 1.2 mg/mL (0.12%)], and Ciloxan 0.3% (Ciprofloxacin) every one hour as bacteria cover. Day 1 post admission, the left BCVA with Pin hole improved to 6/48, IOP was normal, and the conjunctiva injection improved as seen in Fig. 1b. On the 4th day of admission, the patient developed corneal epithelial defect as shown in Fig. 1d. The PCR was positive for VZV and past serology test for VZV indicated Immunoglobulin G (IgG) positive. Infectious diseases and obstetrics consult was offered to patient to rule out anything sinister associated with VZV in pregnancy. Ciloxan was discontinued and eye lubricant six times daily was included. In addition, based on the recommendation of infectious diseases and obstetrics specialists, the topical acyclovir was reinforced with oral acyclovir 400 mg five times daily for 7 days. On the 10th day of admission, unaided visual acuity (VA) improved to 6/6 and the endotheliitis improved significantly as shown in Fig. 1e.

The patient was discharged home and followed up in the out-patient clinic a week, during which her unaided VA was stable at 6/6, and corneal edema including endotheliitis and punctate epithelial erosions (PEEs) resolved leaving behind a faint stromal corneal scar located in peripheral cornea as shown in Fig. 1f. The patient was recommended to taper prednefrin forte and the acyclovir ointment over a 2-week period.

## Discussion

VZV is a highly neurotropic alpha herpesvirus, which has predominantly affected most humans. Primary infection causes chickenpox (varicella) in children, after which virus becomes latent in cranial nerve ganglia, dorsal root ganglia, and autonomic ganglia along the entire neuraxis. VZV may reactivate to cause herpes zoster (shingles), pain, and rash in one to three dermatomes [1]. Ocular manifestations of VZV can include conjunctivitis, uveitis, episcleritis, keratitis, and retinitis [2]. VZV ophthalmic condition is considered an ophthalmologic emergency due to the risk of vision loss if not quickly identified and treated early in the disease course [2]. Few literatures have reported disk-shaped cornea edema associated with endotheliitis in cases of CMV and herpes simplex cornea infection. Lee *et al.* reported a case of coin-shaped corneal endothelial scar in herpes zoster ophthalmicus associated with recurrent anterior uveitis in a 39-year-old Asian female [5]. Unlike Chun and Kim, our case is not a coin-shaped corneal endothelial scar associated with uveitis and herpes zoster ophthalmicus but an isolated disk/coin-shaped corneal edema with endotheliitis without the association of herpes zoster ophthalmicus or uveitis. Like Chun and Kim, we reinforced topical acyclovir 3% five times daily with oral acyclovir 400 mg five times daily in treatment [5]. Whereas topical acyclovir alone may be enough to completely treat epithelial keratitis in herpes zoster, we believe VZV stromal keratitis with endotheliitis would require the addition of oral acyclovir for effective treatment. Porter *et al.* in their study compared local and systemic acyclovir in the management of herpetic disciform keratitis, where they demonstrated that the resolution and improvement of lacrimation and VA was significantly faster and greater in the oral group [6]. Porter *et al.* showed that oral acyclovir (400 mg) taken five times a day is as efficacious as acyclovir (3%) ophthalmic ointment administered five times a day in inhibiting viral replication during the treatment of disciform keratitis with 0.05% prednisolone [6]. In this case, we believe since acyclovir is not teratogenic, adding the oral acyclovir could also serve as prophylaxis against any potential life-threatening systemic diseases associated with VZV in pregnancy [7].
